# Perceptions of Safety of Daily Cannabis vs Tobacco Smoking and Secondhand Smoke Exposure, 2017-2021

**DOI:** 10.1001/jamanetworkopen.2023.28691

**Published:** 2023-08-11

**Authors:** Julia Chambers, Salomeh Keyhani, Pamela M. Ling, Katherine J. Hoggatt, Deborah Hasin, Nhung Nguyen, Anne Woods, Annie Ryder, Beth E. Cohen

**Affiliations:** 1 Department of Medicine, University of California San Francisco; 2 San Francisco Veterans Affairs Health Care System, San Francisco, California; 3 Center for Tobacco Control Research and Education, and Division of General Internal Medicine, University of California San Francisco; 4 Vagelos College of Physicians and Surgeons, Columbia University, New York, New York; 5 Northern California Institute for Research and Education - the Veterans Health Research Institute, San Francisco, California; 6 Department of Psychology, University of Kansas, Lawrence

## Abstract

**Importance:**

While rates of cigarette use are declining, more US adults are using cannabis. Perceptions of safety are important drivers of substance use and public policy; however, little is known about the comparative views of US adults on tobacco and cannabis safety.

**Objective:**

To compare public perceptions of safety of cannabis vs tobacco smoke and evaluate how perceptions may be changing over time.

**Design, Setting, and Participants:**

This longitudinal survey study was conducted using a web-based survey administered in 2017, 2020, and 2021. US adults participating in Ipsos KnowledgePanel, a nationally representative, population-based survey panel, were included. Data were analyzed from March 2021 through June 2023.

**Main Outcomes and Measures:**

Two questions directly compared the perception of safety of cannabis vs tobacco in terms of daily smoking and secondhand smoke exposure. Additional questions assessed perceptions of safety of secondhand tobacco smoke for adults, children, and pregnant women, with an analogous set of questions for secondhand cannabis smoke.

**Results:**

A total of 5035 participants (mean [SD] age, 53.4 [16.2] years; 2551 males [50.7%]) completed all 3 surveys and provided responses for tobacco and cannabis risk questions. More than one-third of participants felt that daily smoking of cannabis was safer than tobacco, and their views increasingly favored safety of cannabis vs tobacco over time (1742 participants [36.7%] in 2017 vs 2107 participants [44.3%] in 2021; *P* < .001). The pattern was similar for secondhand cannabis smoke, with 1668 participants (35.1%) responding that cannabis was safer than tobacco in 2017 vs 1908 participants (40.2%) in 2021 (*P* < .001). Participants who were younger (adjusted odds ratio [aOR] for ages 18-29 years vs ≥60 years, 1.4 [95% CI, 1.1-1.8]; *P* = .01) or not married (aOR, 1.2 [95% CI, 1.0-1.4]; *P* = .01) were more likely to move toward safer views of cannabis use over time, while those who were retired (aOR vs working, 0.8 [95% CI, 0.7-0.9]; *P* = .01) were less likely to move toward a safer view of cannabis. Participants were also more likely to rate secondhand smoke exposure to cannabis vs tobacco as completely or somewhat safe in adults (629 participants [12.6%] vs. 119 participants [2.4%]; *P* < .001), children (238 participants [4.8%] vs. 90 participants [1.8%]; *P* < .001), and pregnant women (264 participants [5.3%] vs. 69 participants [1.4%]; *P* < .001).

**Conclusions and Relevance:**

This study found that US adults increasingly perceived daily smoking and secondhand exposure to cannabis smoke as safer than tobacco smoke from 2017 to 2021. Given that these views do not reflect the existing science on cannabis and tobacco smoke, the findings may have important implications for public health and policy as the legalization and use of cannabis increase.

## Key Points

**Question:**

Is daily smoking of cannabis or exposure to secondhand smoke believed to be safer than tobacco smoking or exposure, and have perceptions changed over time?

**Findings:**

In this survey study of 5035 US adults, daily cannabis smoking or smoke exposure was perceived to be safer than tobacco. Over time, views increasingly favored the safety of cannabis vs tobacco smoke.

**Meaning:**

These findings suggest that public health efforts may be necessary to educate the public on potential risks and curb the increasing social acceptance of cannabis smoke exposure, similar to past education about secondhand tobacco smoke.

## Teaser

This survey study investigates perceptions of the safety of daily cannabis vs tobacco smoking and second hand smoke among US adults from 2017 to 2021.

## Introduction

Decades of federal and state policies and efforts from many nonprofit organizations led to aggressive campaigns to decrease the use of tobacco and nicotine products and exposure to secondhand smoke. These have been credited with dramatically reducing the prevalence of adult cigarette smoking and creating safer smoke-free environments, which in turn, reduce secondhand smoke exposure.^1^ In contrast, there has been increasing legalization and use of cannabis for medicinal and recreational purposes, with rates of adult cannabis use more than doubling from 2001 to 2012.^2^ Although other forms of cannabis are increasing in popularity, smoking is still the most common form.^3^ Studies of cannabis use found that it was associated with multiple negative health outcomes, including cannabis dependence, increased respiratory symptoms, worse cognitive performance, and increased incidence of psychiatric disorders.^4,5,6,7^ Despite this, regulation of cannabis has tended to be less restrictive than that for tobacco, with many smoke-free laws being amended to make exclusions that allow smoking or vaping of cannabis.^8^

Although some studies have found medicinal benefits associated with cannabinoids in treatment of nausea or vomiting from chemotherapy, spasticity related to multiple sclerosis, and refractory epilepsy of childhood, several lines of evidence indicate that cannabis may be harmful and associated with negative health outcomes analogous to those associated with tobacco smoke.^9,10^ Tobacco and cannabis smoke share many chemical compounds that are known carcinogens, and smoking cannabis is associated with increased risk of head and neck and other cancers.^11^ Decades of research on cigarettes and newer research on e-cigarettes has demonstrated that they generate particulate matter that, when inhaled through primary use or secondhand smoke, is associated with increased risk of chronic lung disease and cardiovascular disease.^12,13^ Although less research has been done on cannabis, studies have found that combustion of cannabis, whether through smoking or vaping, produces a greater amount of particulate matter than tobacco, raising concerns that it could be associated with similar health outcomes.^14,15,16,17,18,19^

Given the different trajectories of tobacco and cannabis policy and use, it is important to understand how the perceived safety of daily smoking and secondhand exposure to tobacco and cannabis smoke may be changing. Use of tobacco and cannabis is strongly associated with risk and safety perceptions, and lower risk perception is associated with greater incident and ongoing use of tobacco^20^ and increased use of cannabis.^21,22,23^ Few studies have directly compared the perceived safety of cannabis and tobacco smoke among the same respondents. Most studies assessing risk perception of tobacco and cannabis have looked at the association between use patterns and risk perception for one^7,23,24,25^ or both substances^26,27^ but have not had participants directly compare the 2 substances to each other. In 1 cohort,^27^ cannabis was perceived to be less harmful than tobacco, and other studies have demonstrated that the perception of cannabis has become more favorable over time.^25,26^ As discussed previously, these perceptions are not consistent with published data on potential risks. One study^24^ suggested that risk perception of cannabis may in part be attributed to its relative legality and that as it becomes legalized, the risk perception may decrease further. Understanding the comparative risk perception of tobacco and cannabis is particularly important as it may influence how public health protections and laws enacted for tobacco and electronic nicotine devices are applied in the growing number of states with legal cannabis. Studies have found that many of these states have permitted smoking and vaping of cannabis in settings where tobacco would not be allowed.^8,28^ In addition, a lower comparative risk perception of cannabis may be associated with substitution or increased co-use with tobacco, which could be associated with decreased success in tobacco cessation.^29^

Our study sought to longitudinally examine the perception of safety of daily use of tobacco vs cannabis, as well as secondhand smoke exposure from the 2 substances within a national cohort of US adults. We compared the same respondents’ views on the safety of smoking cannabis and tobacco, as well as secondhand smoke from cannabis and tobacco with respect to populations at increased risk, such as pregnant women and children, at 3 time points from 2017 to 2021. We hypothesized that, over time, US adults would have increasingly favorable views of cannabis compared with tobacco smoke.

## Methods

This survey study follows the American Association for Public Opinion Research (AAPOR) reporting guidance. The Committee of Human Subject Research of the University of California, San Francisco, exempted Ipsos’ conduct of the survey from review because it met criteria outlined in 45 CFR §46.104 (Exempt Research). As an exempt study, this research did not require signed informed consent, but participants were provided information about the study purpose and confidentiality of responses.

### Study Design

Our online survey was conducted in a subset of individuals in Ipsos (formerly GfK) KnowledgePanel, a probability-based, nationally representative panel of the civilian, noninstitutionalized US population^30^ at 3 times to allow for comparison. Ipsos KnowledgePanel uses random, address-based sampling to cover 97% of the US population. Households are provided internet connections and a tablet if needed, and the panel was not an opt-in sample, which reduces sampling and response bias. We stratified our initial sampling by state cannabis legalization status (recreational, medical, or not legal) in 2017.

Questions on the perception of cannabis and tobacco smoke exposure in our survey were created after reviewing questions from existing surveys and the peer-reviewed literature. We also consulted with experts in substance abuse and mental health, cannabis dispensary staff, and cannabis industry professionals. All survey items were written at an eighth-grade reading level, and comprehension testing was conducted in a sample of 40 adults of different ages, educational levels, and cannabis and tobacco use statuses. Subsequent waves of the survey were piloted internally by the study team and by Ipsos on a random sample of panel participants to review and refine online administration. Full details of baseline survey design and administration were previously published.^31^

The first wave was administered from September 27, 2017, to October 9, 2017, to 16 280 US adults, with 9003 people completing the survey (response rate, 55.3%). The second wave of the survey, from August 5 to 31, 2020, was administered to 8529 respondents of the original survey who remained available for contact, with responses from 5979 participants (66.4% of the original cohort, 70.1% of individuals contacted). The third wave of the survey was administered from August 5, 2021, to August 31, 2021, to the 7305 respondents from the first or second wave of the survey, with responses from 5420 participants (60.2% of the original cohort, 74.2% of individuals contacted). A total of 5053 people responded to all 3 waves of the survey. Of these, 18 individuals did not respond to any tobacco or cannabis risk questions and were therefore excluded from the study, leaving an analytic sample of 5035 participants (eFigure in Supplement 1). Individuals who completed the baseline survey but did not respond to the second or third wave were significantly more likely to be younger, members of racial or ethnic minority groups (Black, Hispanic, multiracial [participants who chose >1 racial option], and other [including American Indian or Alaska Native, Asian, Native Hawaiian or Other Pacific Islander, or a different race response ]), have lower educational attainment, and report current use of tobacco or cannabis at baseline (eTable 1 in Supplement 1). Nonrespondents were significantly more likely at baseline to view cannabis smoke as being safer than tobacco smoke and to hold safer views of secondhand cannabis smoke exposure (eTable 1 in Supplement 1).

### Measures

The survey used the term “marijuana,” and participants were advised this referred to “cannabis, pot, weed, grass, and hash.” To directly compare views on the safety of smoking cannabis and tobacco, we asked, “How does smoking one marijuana joint a day compare with smoking one cigarette a day?” with response options of “Smoking one marijuana joint a day is much less safe, somewhat less safe, as safe as, somewhat safer, or much safer than smoking one cigarette a day.” To directly compare views on safety of secondhand smoke, we asked, “How does secondhand smoke from marijuana compare to secondhand smoke from tobacco?” with response options of “Secondhand smoke from marijuana is much less safe, somewhat less safe, as safe as, somewhat safer, or much safer than secondhand smoke from tobacco.” Both questions were used in all 3 waves of the survey.

To compare views on safety of secondhand smoke exposure for specific populations, we asked, “How safe is it to expose [children, pregnant women, adults] to secondhand smoke from marijuana?” We asked analogous questions with “tobacco” instead of “marijuana.” Response options for all 6 questions were “completely unsafe,” “somewhat unsafe,” “somewhat safe,” and “completely safe.” Questions about secondhand tobacco smoke were included in only 2020 and 2021 waves.

To reduce systematic error, we asked questions about secondhand smoke for tobacco vs cannabis in the same way. Finally, we attempted to reduce response bias with nonnegative, unbiased language in the survey. Many respondents in the pilot of the survey did not have a negative view of cannabis, and so we chose to use the words “safe” and “safer” rather than “risk” to avoid introducing bias into our study population.^31,32^

### Independent Variables

Participant demographics, including age, sex, race, ethnicity, education level, annual income, employment status, marital status, and state of residence, were obtained by Ipsos via self-report. Race and ethnicity were assessed separately and combined into a single variable. Ethnicity was assessed by asking, “Are you of Hispanic, Latino, or Spanish origin?” Individuals responding yes were coded as Hispanic in the combined variable for all race responses. Those responding no were coded as non-Hispanic Black, non-Hispanic White, non-Hispanic other race, or non-Hispanic multiracial (ie, respondents who chose multiple race options) based on their response to the race question. Non-Hispanic other race includes American Indian or Alaska Native, Asian, Native Hawaiian or Other Pacific Islander, or a different race response for the race question. The combined race and ethnicity variable and its categories were created by Ipsos to match the US Census Bureau’s Current Population Survey benchmarks, which are used in survey weighting. State cannabis legal status in each year was determined as not legal, medical legal, or recreational legal based on laws that had been enacted by the survey date. We classified participants as having a change in cannabis legal status if their state of residence changed from not legal to medical or recreational legal or from medical to recreational legal over the study period. We also classified participants as having a change in legal status if they moved to a state that had greater legal access (medical or recreational vs not legal or recreational vs medical legal) during the study period.

### Statistical Analysis

To evaluate changes in views over time in questions that directly compared the safety of primary and secondhand cannabis vs tobacco exposure, we compared the proportion of respondents endorsing each response option in 2017 vs 2021 using multinomial logistic regression models. Survey year was entered as a covariate, and we used a variance estimator that accounts for repeated measures within individuals in the longitudinal survey cohort. We identified individuals who from 2017 to 2021 moved toward a safer view of cannabis vs tobacco across the 5 response options for each question (for example, moving from a response that secondhand smoke from cannabis was “somewhat safer” to “much safer” than tobacco). Individuals who rated cannabis as “much safer” than tobacco in 2017 were excluded from analyses given that they had already endorsed the most favorable view of cannabis. We used χ^2^ tests and *t* tests as appropriate to compare baseline characteristics from Table 1 for individuals whose views increasingly favored cannabis over time vs those whose views were unchanged or who increasingly favored tobacco (eTable 2 in Supplement 1). We then developed multivariate logistic regression models with this dichotomous dependent variable (cannabis safer vs less safe or no change) that included as independent variables all factors associated with the outcome at a significance level of *P* ≤ .10 in univariate analyses. We used the Homer-Lemeshow test for goodness of fit, which yielded values of *P* > .9, indicating good fit. We repeated these analyses using multivariate linear regression models with a continuous dependent variable representing the change in views from 2017 to 2021. For this outcome, we treated Likert scale responses as values of 1 (tobacco much safer) to 5 (cannabis much safer) and calculated the change as the value in 2021 minus the value in 2017. We performed model diagnostics for linear regression models and did not identify violations of key modeling assumptions.

**Table 1.** Baseline Characteristics of Respondents

Characteristic	Respondents, No. (%) (N = 5035)
Age mean (SD), y	53.4 (16.2)
Sex	
Female	2484 (49.3)
Male	2551 (50.7)
Race and ethnicity	
Black, non-Hispanic	314 (6.2)
Hispanic	447 (8.9)
White, non-Hispanic	3945 (78.4)
Multiracial, non-Hispanic^a^	127 (2.5)
Other, non-Hispanic^b^	202 (4.0)
Education	
<High school	194 (3.9)
High school	1070 (21.3)
Some college	1508 (30.0)
≥Bachelor’s degree	2263 (45.0)
Income, $	
$30 000	839 (16.7)
30 000-74 999	1728 (34.3)
75 000-124 999	1403 (27.9)
$125 000	1065 (21.2)
Marital status	
Married	3111 (62.0)
Other	1914 (38.0)
Working status	
Retired	1399 (27.8)
Working	3014 (59.9)
Other	622 (12.4)
Current tobacco smoking or vaping^c^	523 (10.5)
Cannabis use past 30 d	392 (7.8)
State cannabis legal status	
Not legal	1659 (33.0)
Medical legal	1999 (39.7)
Recreational legal	1377 (27.4)
State change in legal status^c^	
No change	3363 (67.1)
State changed legal status	1537 (30.6)
Moved to a state with different legal status	115 (2.3)

^a^ Multiracial was used for participants who self-selected more than 1 racial category.

^b^ Non-Hispanic other race includes American Indian or Alaska Native, Asian, Native Hawaiian or Other Pacific Islander, or a different race response for the race question.

^c^ Data were missing for 31 respondents for current tobacco smoking or vaping and 20 respondents for state change in cannabis legal status.

To evaluate changes over time in views on safety of secondhand cannabis or tobacco smoke in specific populations (adults, children, and pregnant women), we compared the proportion of respondents endorsing each response option in 2021 to 2017 (for cannabis questions) and 2020 (for tobacco questions) using multinomial logistic models with a variance estimators to account for repeated measures. We repeated analyses applying survey weights provided by Ipsos that accounted for study design and nonresponse. Analyses were conducted with SAS Enterprise statistical software version 7.1 (SAS Institute) or Stata/SE statistical software version 14 (StataCorp). All tests used a 2-sided α < .05 for statistical significance. Data were analyzed from March 2021 through June 2023.

## Results

There were 5035 survey respondents (mean [SD] age, 53.4 [16.2] years; 2551 males [50.7%]; 314 Black [6.2%], 447 Hispanic [8.9%], and 3945 White [78.4%]) with data on risk perceptions of tobacco and cannabis smoke in all 3 years (2017, 2020, and 2021) (Table 1). Less than half of respondents had a bachelor’s degree or higher (2263 individuals [45.0%]), and the largest proportion of people had an income of $30 000 to $74 999 (1728 individuals [34.3%]). Most respondents were married (3111 individuals [62.0%]) and employed (3014 individuals [59.9%]).

### Perception of Safety of Daily Smoking of Tobacco or Cannabis

Regarding the safety of daily smoking of cannabis vs tobacco, there was a significant shift from 2017 to 2021 toward a more favorable perception of cannabis. In 2021 compared with 2017, there were fewer people reporting that cannabis was somewhat or much less safe than tobacco (1213 participants [25.5%] vs 1601 participants [33.7%]) and more people reporting that cannabis was somewhat safer or much more safe than tobacco (2107 participants [44.3%] vs 1742 participants [36.7%]) (*P* < .001). (Table 2). Weighted analyses yielded similar findings (eTable 3 in Supplement ).

**Table 2.** Change in Views on Safety of Exposure to Cannabis vs Tobacco

Question	Respondents, No. (%)			Change from 2021-2017, No. (%)	*P* value
	2017	2020	2021		
**How safe is smoking one marijuana joint a day vs one cigarette a day? (n = 4751)**					
One marijuana joint much less safe than one cigarette	783 (16.5)	634 (13.3)	630 (13.2)	−153 (−3.3)	<.001
One marijuana joint somewhat less safe than one cigarette	818 (17.2)	612 (12.9)	583 (12.3)	−235 (−4.9)	
One marijuana joint as safe as smoking one cigarette	1408 (29.6)	1433 (30.1)	1431 (30.1)	23 (0.5)	
One marijuana joint somewhat safer than one cigarette	976 (20.5)	1340 (28.2)	1379 (29.0)	403 (8.5)	
One marijuana joint much safer than one cigarette	766 (16.1)	732 (15.4)	728 (15.3)	−38 (−0.8)	
**How does secondhand smoke from marijuana compare to secondhand smoke from tobacco? (n = 4749)**					
Secondhand smoke from marijuana much less safe than from tobacco	705 (14.8)	554 (11.7)	566 (11.9)	−139 (−2.9)	<.001
Secondhand smoke from marijuana is somewhat less safe than from tobacco	685 (14.4)	515 (10.8)	527 (11.1)	−158 (−3.3)	
Secondhand smoke from marijuana is as safe as from tobacco	1691 (35.6)	1753 (36.9)	1748 (36.8)	57 (1.2)	
Secondhand smoke from marijuana is somewhat safer than from tobacco	1029 (21.7)	1324 (27.9)	1324 (27.9)	295 (6.2)	
Secondhand smoke from marijuana is much safer than from tobacco	639 (13.4)	577 (12.1)	584 (12.2)	−55 (−1.2)	

### Secondhand Smoke Exposure

Findings for questions directly comparing the safety of secondhand smoke exposure to cannabis vs tobacco were similar to questions about daily smoking, with a significant shift toward a more favorable view of cannabis from 2017 to 2021. In 2021 compared with 2017, there were fewer people reporting that secondhand smoke was somewhat or much less safe for cannabis vs tobacco (1093 participants [23.0%] vs 1390 participants [29.3%]) and more individuals responding that secondhand smoke was somewhat safer or much more safe for cannabis vs tobacco (1908 participants [40.2%] vs 1668 participants [35.1%]) (*P* < .001) (Table 2). Findings in weighted analyses were similar (eTable 3 in Supplement 1).

### Secondhand Smoke Exposure in Specific Groups

Perceptions on the safety of exposure to secondhand smoke from cannabis and tobacco for children, adults, and pregnant women remained similar over the study period (eTable 4 in Supplement 1). However, at all times, more people endorsed the greater safety of secondhand smoke exposure for cannabis vs tobacco in all groups; these differences were most dramatic for exposure of adults (Figure). Participants were more likely to rate secondhand smoke exposure to cannabis vs tobacco as completely or somewhat safe in adults (629 participants [12.6%] vs 119 participants [2.4%]; *P* < .001), children 238 participants [4.8%] vs 90 participants [1.8%]; P < .001), and pregnant women (264 participants [5.3%] vs 69 participants [1.4%]; P < .001). Findings were not substantially changed in weighted analyses (eTable 5 in Supplement 2).

**Figure.**
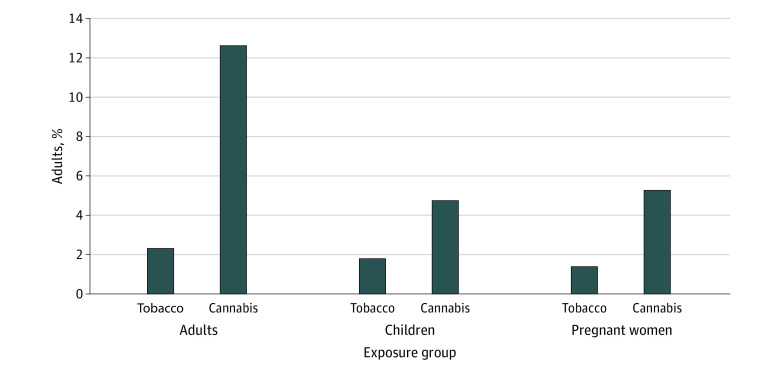
Proportion of US Adults Viewing Tobacco vs Cannabis Secondhand Smoke Exposure as Safe The proportion of adults who viewed tobacco vs cannabis secondhand smoke exposure as somewhat or completely safe for adults and populations at increased risk is presented.

### Factors Associated With Favorable Change in Cannabis Risk Perception

As an additional analysis, we looked at factors associated with a favorable change in cannabis vs tobacco risk perception over time (Table 3). Younger individuals (adjusted odds ratio [aOR] for ages 18-29 years vs ≥60 years, 1.4 [95% CI, 1.1-1.8]; *P* = .01) and those who were not married (aOR, 1.2 [95% CI, 1.0-1.4]; *P* = .01) were significantly more likely to move toward viewing smoking cannabis as safer than cigarettes, while those who were retired (aOR vs working, 0.8 [95% CI, 0.7-0.9]; *P* = .01) were less likely to move in this direction (Table 3). In analyses focused on secondhand smoke, individuals who were retired were less likely to shift toward perceiving cannabis secondhand smoke as safer compared with tobacco secondhand smoke over time (aOR, 0.8 [95% CI, 0.6-0.9]; *P* = .007) (Table 3). Weighted analyses yielded similar findings except that in analyses of secondhand smoke perceptions, working status was no longer associated with a change in outcome (eTable 6 in Supplement 1). Sensitivity analyses using a continuous outcome for change yielded similar findings (eTable 7 in Supplement 1), with the same factors associated with movements toward more or less safe views of cannabis vs tobacco smoke. In these linear models, female sex was also associated with moving toward a safer view of exposure to primary cannabis smoke.

**Table 3.** Characteristics Associated with Change in Perception of Exposure to Marijuana as Safer

Characteristic	Adjusted OR (95% CI)	*P* value
**How safe is smoking one marijuana joint a day vs one cigarette a day? (n = 3985)**		
Age, y		
18-29	1.4 (1.1-1.8)	.01
30-44	1.1 (0.9-1.3)	.61
45-59	1.2 (1.0-1.4)	.07
≥60	1 [Reference]	NA
Martial status		
Married	1 [Reference]	NA
Other	1.2 (1.0-1.4)	.01
Working status		
Working	1 [Reference]	NA
Retired	0.8 (0.7-0.9)	.01
Other	1.1 (0.8-1.2)	.59
State baseline legal status		
Not legal	1 [Reference]	NA
Medical legal	1.1 (0.9-1.2)	.39
Recreational legal	0.9 (0.8-1.1)	.26
**How does secondhand smoke from marijuana compare to secondhand smoke from tobacco? (n = 3985)**		
Age, y		
18-29	1.1 (0.8-1.4)	.57
30-44	0.9 (0.7-1.1)	.16
45-59	1.0 (0.8-1.2)	.97
≥60	1 [Reference]	NA
Working status		
Working	1 [Reference]	NA
Retired	0.8 (0.6-0.9)	.007
Other	1.0 (0.8-1.3)	.77
State baseline legal status		
Not legal	1 [Reference]	NA
Medical legal	1.1 (1.0-1.3)	.09
Recreational legal	1.0 (0.8-1.1)	.67

## Discussion

In this national longitudinal survey study, we found that individuals perceived daily use and secondhand smoke exposure as safer for cannabis than tobacco and that the perceived relative safety of cannabis smoke increased over time. Respondents who were younger and unmarried had an increasingly safe perception of smoking cannabis vs tobacco over time. However, legality of cannabis in the participants’ state of residence was not independently associated with change over time. This suggests that the increasing perception of safety of cannabis may be a larger, national trend rather than a trend seen only in states with cannabis legalization. More participants at each time believed secondhand smoke from cannabis vs tobacco was somewhat or completely safe. This difference was most notable for exposure in adults but apparent even in populations at greater risk, such as pregnant women and children. These views did not substantially change over our study period.

Our study expands upon prior work on public opinions about the safety of cannabis and tobacco. The National Survey on Drug Use and Health found that the proportion of respondents who felt that regular cannabis use was associated with great risk decreased from 51.3% in 2002 to 40.3% in 2012.^33^ Our study found that these trends continued in the era of recreational cannabis legalization. Few studies have simultaneously assessed tobacco and cannabis risk perception, and risk has typically been measured separately^26,27^ rather than by asking participants to directly compare substances. Studies that have looked at both tobacco and cannabis assessed risk perception and use in samples from Australia (1046 participants)^27^ and Germany (318 participants)^26^ and found that lower risk perception of tobacco and cannabis was associated with concurrent and future use.

As cannabis becomes legalized in more states, risk perception may decrease further, which may be associated with increased consumption of cannabis and exposure to secondhand cannabis smoke. These cultural shifts toward increasingly safer views of cannabis may be partly attributable to the $17.5 billion legal cannabis industry’s marketing, which often highlights unsubstantiated safety claims and health benefits^34,35^ without noting existing data on potential harms associated with smoking and vaping, many of which are similar to those associated with tobacco. In 1 study,^36^ internet claims about the health benefits of cannabis were found to have evidence of support in 5% of cases. Although there are some moderate-quality data to support the use of cannabis in specific health circumstances (eg, spasticity), there are many other conditions that social media and the cannabis industry claim cannabis can improve despite a lack of scientific evidence.^6,36^

More research is needed to investigate the potential medicinal benefits associated with cannabis, but this must be balanced by investigation of potential negative health outcomes, particularly for smoked or vaped cannabis products. Animal studies suggest that even 1 minute of secondhand smoke from cannabis may be associated with impeded endothelial function and therefore the same cardiovascular outcomes as tobacco.^19^ Smoking cannabis is associated with increased risk of head or neck and other cancers.^11^ Tobacco and cannabis smoke share many chemical compounds that are characterized as carcinogens.^11^ There is also moderate-quality evidence to suggest that cannabis use, especially heavy use, is associated with a higher risk of psychiatric conditions, including psychosis,^37^ depression,^38^ and anxiety^39^. Lower-quality data suggest there is a higher risk of respiratory side effects with cannabis use,^40^ but more studies are needed to fully understand associations with cardiovascular health^41^ and lung, oral, and other cancers.^42^

Understanding changing views on tobacco and cannabis risk is important given that increases in social acceptance and decreases in risk perception may be directly associated with public health and policies. For example, there is a movement to make exceptions for cannabis in smoke-free bans in public housing, bars, and restaurants, although there are no data that suggest that secondhand exposure is safe.^8,43^ In fact, emerging data suggest significant potential risks associated with exposure to secondhand cannabis smoke.^14^ Such smoke-free air laws are important for protecting public health, especially among already high-risk communities, and have been shown to be associated with reduced youth smoking initiation.^1,44^ Exceptions for cannabis use may also erode the effectiveness of tobacco smoking bans and still leave the public at risk from harmful compounds present in cannabis.^11^ Finally, many products combine cannabis with tobacco, and the substances are used in similar or identical devices, making enforcement of tobacco bans challenging.^45^

### Limitations

Although our study benefits from a longitudinal design and national sample, our findings should be interpreted in light of several limitations. The KnowledgePanel attempts to avoid intrinsic biases of many online surveys by using national, representative sampling and not allowing opt-in volunteers, but the generalizability of this study may be limited by nonresponse and loss to follow-up over time. The wording of questions in the survey may have also introduced bias in respondents, although our intent was not to do so. In addition, we asked specifically about safety of smoking cannabis joints vs tobacco cigarettes and cannot compare perceptions of safety of the many other forms of smoked and vaped cannabis, tobacco, and nicotine.

## Conclusions

This survey study found that despite a lack of data, US adults have a more favorable view of the safety of primary and secondhand cannabis smoke exposure than tobacco smoke exposure. As the cannabis industry and use of cannabis continue to grow, these risk perceptions may further decrease and have the potential to shape public policy. This may have serious health implications at individual and societal levels. Thus, more research is necessary to understand the potential risks associated with cannabis use to plan a public health and regulatory response.

## Notes

Supplement 1. **eFigure.** Study Cohort Construction **eTable 1.** Baseline Characteristics of Respondents to Survey Waves vs Nonrespondents **eTable 2.** Univariate Analyses Showing Factors Associated With a Change in Perception From 2017 to 2021 of Primary and Secondhand Smoke Exposure to Cannabis as Safer Than Tobacco **eTable 3.** Weighted Analyses of Change in Views on Safety of Primary and Secondhand Smoke Exposure to Cannabis vs Tobacco **eTable 4.** Analogous Questions on Safety of Secondhand Smoke Exposure From Tobacco or Cannabis **eTable 5.** Weighted Analyses for Analogous Question on Safety of Secondhand Smoke Exposure From Tobacco or Cannabis **eTable 6.** Weighted Multivariable Analysis of Baseline Characteristics Associated With Change in Perception of Primary and Secondhand Smoke Exposure to Marijuana as Safer **eTable 7.** Multivariable Linear Analysis of Baseline Characteristics Associated With Change in Perception of Primary and Secondhand Smoke Exposure

Supplement 2.
Data Sharing Statement
