# Supplementary material for: Perceptions of Safety of Daily Cannabis vs Tobacco Smoking and Secondhand Smoke Exposure, 2017-2021
Source: JAMA Netw Open. 2023 Aug 11;6(8):e2328691. doi: 10.1001/jamanetworkopen.2023.28691 (PMC10422186; doi:10.1001/jamanetworkopen.2023.28691)
Supplement: Supplement 1. — eFigure. Study Cohort Construction eTable 1. Baseline Characteristics of Respondents to Survey Waves vs Nonrespondents eTable 2. Univariate Analyses Showing Factors Associated With a Change in Perception From 2017 to 2021 of Primary and Secondhand Smoke Exposure to Cannabis as Safer Than Tobacco eTable 3. Weighted Analyses of Change in Views on Safety of Primary and Secondhand Smoke Exposure to Cannabis vs Tobacco eTable 4. Analogous Questions on Safety of Secondhand Smoke Exposure From Tobacco or Cannabis eTable 5. Weighted Analyses for Analogous Question on Safety of Secondhand Smoke Exposure From Tobacco or Cannabis eTable 6. Weighted Multivariable Analysis of Baseline Characteristics Associated With Change in Perception of Primary and Secondhand Smoke Exposure to Marijuana as Safer eTable 7. Multivariable Linear Analysis of Baseline Characteristics Associated With Change in Perception of Primary and Secondhand Smoke Exposure [file jamanetwopen-e2328691-s001.pdf]

Supplemental Online Content

Chambers J, Keyhani S, Ling PM, et al. Perceptions of safety of daily cannabis vs tobacco smoking and secondhand smoke exposure, 2017-2021. *JAMA Netw Open*. 2023;6(8):e2328691. doi:10.1001/jamanetworkopen.2023.28691

eFigure. Study Cohort Construction

eTable 1. Baseline Characteristics of Respondents to Survey Waves vs Nonrespondents

eTable 2. Univariate Analyses Showing Factors Associated With a Change in Perception From 2017 to 2021 of Primary and Secondhand Smoke Exposure to Cannabis as Safer Than Tobacco

eTable 3. Weighted Analyses of Change in Views on Safety of Primary and Secondhand Smoke Exposure to Cannabis vs Tobacco

eTable 4. Analogous Questions on Safety of Secondhand Smoke Exposure From Tobacco or Cannabis

eTable 5. Weighted Analyses for Analogous Question on Safety of Secondhand Smoke Exposure From Tobacco or Cannabis

eTable 6. Weighted Multivariable Analysis of Baseline Characteristics Associated With Change in Perception of Primary and Secondhand Smoke Exposure to Marijuana as Safer

eTable 7. Multivariable Linear Analysis of Baseline Characteristics Associated With Change in Perception of Primary and Secondhand Smoke Exposure

This supplemental material has been provided by the authors to give readers additional information about their work.

© 2023 Chambers J et al. *JAMA Network Open.*

eFigure 1: Study Cohort Construction

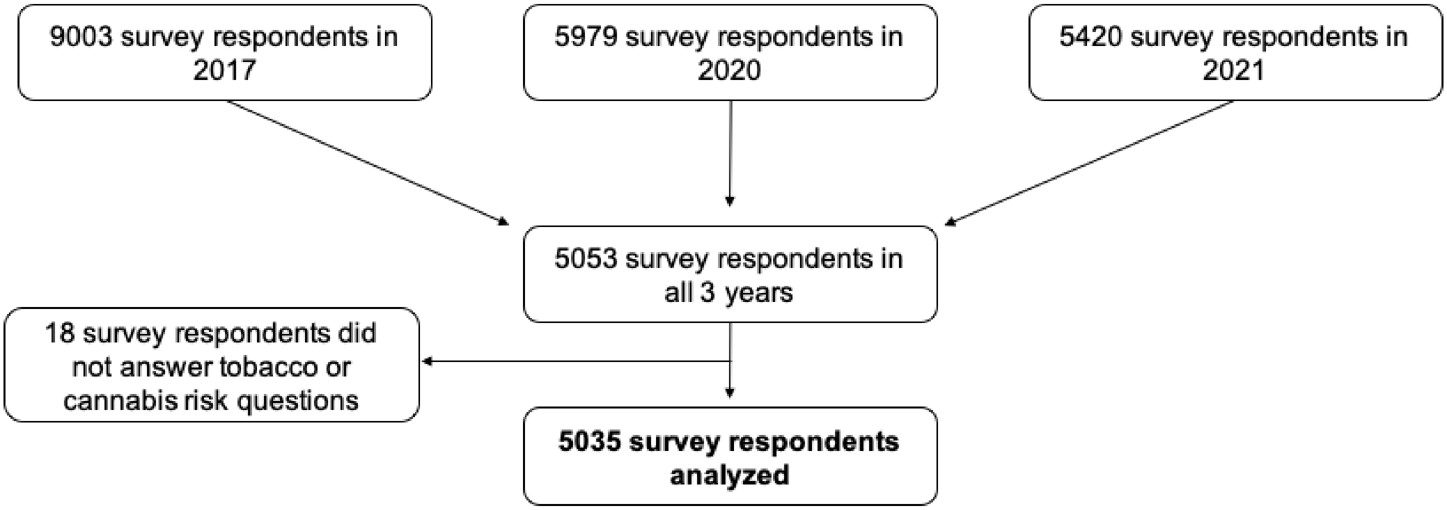

© 2023 Chambers J et al. *JAMA Network Open.*

eTable 1: Baseline Characteristics of Respondents to All Three Survey Waves vs Nonrespondents*

	Respondents (n = 5053)	Non-respondents (n=3950)	p-value
**Age, y ± SD**	53.4 ± 16	51.9 ± 18	
**Gender, n (%)**			<.001
Female	2491 (49.3)	2205 (55.8)	
Male	2562 (50.7)	1745 (44.2)	
**Race/Ethnicity, n (%)**			<.001
Black, non-Hispanic	317 (6.3)	349 (8.8)	
Hispanic	448 (8.9)	470 (11.9)	
Multiracial, non-Hispanic	128 (2.5)	119 (3.0)	
White, non-Hispanic	3957 (78.3)	2871 (72.7)	
Other, non-Hispanic	203 (4.0)	141 (3.6)	
**Education, n (%)**			<.001
Less than high school	194 (3.8)	254 (6.4)	
High school	1076 (21.3)	953 (24.1)	
Some college	1513 (29.9)	1355 (34.3)	
Bachelor's degree or higher	2270 (44.9)	1388 (35.1)	
**Income, n (%)**			<.001
<$30,000	842 (16.7)	933 (23.6)	
$30,000-74,999	1731 (34.2)	1441 (36.5)	
$75,000-124,999	1412 (27.9)	929 (23.5)	
>$125,000	1068 (21.1)	647 (16.4)	
**Marital Status, n (%)**			<.001
Married	3131 (62.0)	2194 (55.5)	
Other	1922 (38.0)	1756 (44.5)	
**Working status, n (%)**			<.001
Retired	1403 (27.8)	1026 (26.0)	
Working	3026 (59.9)	2283 (57.8)	
Other	624 (12.4)	641 (16.2)	
**Current tobacco smoking/vaping, n (%)**	523 (10.4)	609 (15.6)	<.001
**Cannabis use past 30 days, n (%)**	392 (7.8)	389 (9.9)	<.001
**Baseline state cannabis legal status n (%)**			.07
Non-legal	1667 (33.0)	1392 (35.2)	
Medical legal	2008 (39.7)	1534 (38.8)	
Recreational legal	1378 (27.3)	1024 (25.9)	
**How safe is smoking one marijuana joint a day versus one cigarette a day?**			<.001
One marijuana joint much less safe than one cigarette	828 (16.9)	688 (17.9)	
One marijuana joint somewhat less safe than one cigarette	844 (17.2)	585 (15.3)	
One marijuana joint as safe as smoking one cigarette	1448 (29.6)	1046 (27.3)	
One marijuana joint somewhat safer than one cigarette	996 (20.3)	746 (19.4)	
One marijuana joint much safer than one cigarette	780 (15.9)	772 (20.1)	

© 2023 Chambers J et al. *JAMA Network Open.*

**How does secondhand smoke from marijuana compare to secondhand smoke from tobacco?**			<.001
Secondhand smoke from marijuana much less safe than from tobacco	759 (15.5)	627 (16.3)	
Secondhand smoke from marijuana is somewhat less safe than from tobacco	714 (14.6)	542 (14.1)	
Secondhand smoke from marijuana is as safe as from tobacco	1733 (35.3)	1192 (31.0)	
Secondhand smoke from marijuana is somewhat safer than from tobacco	1050 (21.4)	846 (21.9)	
Secondhand smoke from marijuana is much safer than from tobacco	648 (13.2)	640 (16.6)	

*Missing data: Current tobacco smoking/vaping, n=31; State change in legal status, n=20; Safety of smoking marijuana vs. tobacco, n=270; Safety of secondhand marijuana vs. tobacco smoke, n=252.
*Note: Table 1 in the main manuscript excludes non-respondents plus 18 participants who responded to all three waves of the survey but did not answer cannabis/tobacco risk questions.

© 2023 Chambers J et al. *JAMA Network Open.*

eTable 2: Univariate Analyses Showing Factors Associated with a Change in Perception from 2017 to 2021 of Primary and Secondhand Smoke Exposure to Cannabis as Safer than Tobacco

How safe is smoking one marijuana joint a day versus one cigarette a day? n =3985*			
	**Views did not move towards safer**	**Views moved towards safer**	**p-value**
**Age**			.005
18-29	222 (55.1)	181 (44.9)	
30-44	425 (60.5)	277 (39.5)	
45-59	685 (58.1)	494 (41.9)	
60+	1119 (65.8)	582 (34.2)	
**Gender**			.27
Female	1265 (62.3)	764 (37.7)	
Male	1186 (60.6)	770 (39.7)	
**Race/Ethnicity, n (%)**			.87
Black, non-Hispanic	138 (61.1)	88 (38.9)	
Hispanic	192 (60.6)	125 (39.4)	
Multiracial, non-Hispanic	52 (59.1)	36 (40.9)	
White, non-Hispanic	1959 (61.5)	1226 (38.5)	
Other, non-Hispanic	110 (65.1)	59 (34.9)	
**Education**			.92
High school	77 (61.1)	49 (38.9)	
Some college	489 (60.4)	320 (39.6)	
Bachelor's degree or higher	729 (61.8)	450 (38.2)	
Less than high school	1156 (61.8)	715 (38.2)	
**Income**			.30
<$30,000	384 (64.2)	214 (35.8)	
$30,000-74,999	838 (62.0)	514 (38.0)	
$75,000-124,999	693 (61.2)	440 (38.3)	
>$125,000	536 (59.4)	366 (40.6)	
**Marital**			.09
Other	898 (63.3)	521 (36.7)	
Married	1553 (60.5)	1013 (39.5)	
**Working status**			<.001
Retired	788 (67.5)	379 (32.5)	
Other	257 (57.8)	188 (42.3)	
Working	1406 (59.3)	967 (40.8)	
**Current tobacco/nicotine use**			.48
Yes	234 (63.2)	136 (36.8)	
No	2202 (61.4)	1387 (38.7)	
**Marijuana in the last 30 days**			.48
Yes	114 (64.0)	64 (36.0)	
No	2337 (61.4)	1470 (38.6)	
**State cannabis legal status**			.01
Non-legal	806 (60.7)	522 (39.3)	
Medical legal	921 (59.7)	623 (40.4)	
Recreational legal	724 (65.0)	389 (35.0)	
**State change in legal status**			
State law change	775 (59.9)	518 (40.1)	.15
State law did not change	1668 (62.3)	1008 (37.7)	
**State change in legal status**			
Moved to state with different status	52 (57.1)	39 (42.9)	.38

© 2023 Chambers J et al. *JAMA Network Open.*

Did not move to state with different status	2391 (61.6)	1487 (38.4)	
**How does secondhand smoke from marijuana compare to secondhand smoke from tobacco? n=4,110***			
	**Views did not move towards safer**	**Views moved towards safer**	**p-value**
**Age**			.04
18-29	266 (60.9)	171 (39.1)	
30-44	481 (66.7)	240 (33.3)	
45-59	778 (63.6)	445 (36.4)	
60+	1158 (67.0)	571 (33.0)	
**Gender**			.98
Female	1326 (65.3)	706 (34.7)	
Male	1357 (65.3)	721 (34.7)	
**Race/Ethnicity, n (%)**			.21
Black, non-Hispanic	139 (59.1)	96 (40.9)	
Hispanic	213 (65.1)	114 (34.9)	
Multiracial, non-Hispanic	68 (70.8)	28 (29.2)	
White, non-Hispanic	2145 (65.4)	1134 (34.6)	
Other, non-Hispanic	118 (68.2)	55 (31.8)	
**Education**			.13
High school	76 (59.4)	52 (40.6)	
Some college	524 (63.3)	304 (36.7)	
Bachelor's degree or higher	788 (64.8)	428 (35.2)	
Less than high school	1295 (66.8)	643 (33.2)	
**Income**			.89
<$30,000	401 (65.1)	215 (34.9)	
$30,000-74,999	905 (64.6)	497 (35.5)	
$75,000-124,999	772 (66.0)	398 (34.0)	
>$125,000	605 (65.6)	317 (34.4)	
**Marital**			.69
Other	976 (65.7)	510 (34.3)	
Married	1707 (65.1)	917 (35.0)	
**Working status**			.004
Retired	813 (69.2)	362 (30.8)	
Other	287 (63.1)	168 (36.9)	
Working	1583 (63.8)	897 (36.2)	
**Current tobacco/nicotine use**			.37
Yes	255 (67.5)	123(32.5)	
No	2414 (65.1)	1292 (34.9)	
**Marijuana in the last 30 days**			.74
Yes	131 (64.2)	73 (35.8)	
No	2552 (65.3)	1354 (34.7)	
**State cannabis legal status**			
Non-legal	894 (66.1)	459 (33.9)	.04
Medical legal	1011 (63.0)	593 (37.0)	
Recreational legal	778 (67.5)	375 (32.5)	
**State change in legal status**			
State law change	855 (63.7)	488 (36.3)	.13
State law did not change	1819 (66.1)	933 (33.9)	
**State change in legal status**			
Moved to state with different status	59 (61.5)	37 (38.5)	.42

© 2023 Chambers J et al. *JAMA Network Open.*

Did not move to state with different status	2615 (65.4)	1384 (34.6)

*Analyses exclude participants that already had the safest view of cannabis at baseline

© 2023 Chambers J et al. *JAMA Network Open.*

eTable 3: Weighted Analyses of Change in Views on Safety of Primary and Secondhand Smoke Exposure to Cannabis vs. Tobacco

How safe is smoking one marijuana joint a day versus one cigarette a day?					
	**2017**	**2020**	**2021**	**2021-2017**	**p-value**
	% (95% CI)	% (95% CI)	% (95% CI)	Δ%	<.001
One marijuana joint much less safe than one cigarette	17.8 (16.4 – 19.4)	13.9 (12.9 – 15.3)	14.0 (12.7 – 15.4)	-3.8	
One marijuana joint somewhat less safe than one cigarette	15.8 (14.5 – 17.2)	11.1 (10.0 – 12.3)	10.5 (9.4 – 11.7)	-5.3	
One marijuana joint as safe as smoking one cigarette	26.5 (24.8 – 28.2)	27.4 (25.8 – 29.2)	27.4 (25.7 – 29.1)	0.9	
One marijuana joint somewhat safer than one cigarette	21.3 (19.8 – 23.0)	29.3 (27.5 – 31.1)	30.0 (28.3 – 31.9)	8.7	
One marijuana joint much safer than one cigarette	18.6 (17.0 – 20.2)	18.3 (16.8 – 19.9)	18.1 (16.5 – 19.7)	-0.5	

How does secondhand smoke from marijuana compare to secondhand smoke from tobacco?					
	**2017**	**2020**	**2021**	**2021-2017**	**p-value**
	% (95% CI)	% (95% CI)	% (95% CI)	Δ%	
Secondhand smoke from marijuana much less safe than from tobacco	16.5 (15.1 – 18.0)	12.3 (11.1 – 13.7)	12.2 (11.0 – 13.6)	-4.3	<.001
Secondhand smoke from marijuana is somewhat less safe than from tobacco	14.1 (12.8 – 15.5)	10.1 (9.0 – 11.3)	10.5 (9.4 – 11.8)	-3.6	
Secondhand smoke from marijuana is as safe as from tobacco	31.3 (29.5 – 33.1)	33.8 (32.0 – 35.7)	34.1 (32.3 – 36.0)	2.8	
Secondhand smoke from marijuana is somewhat safer than from tobacco	22.0 (20.4 – 23.6)	29.9 (28.1 – 31.8)	28.9 (27.1 – 30.7)	6.9	
Secondhand smoke from marijuana is much safer than from tobacco	16.2 (14.7 – 17.8)	13.9 (12.5 – 15.3)	14.3 (12.9 – 15.8)	-1.9	

© 2023 Chambers J et al. *JAMA Network Open.*

eTable 4: Analogous Questions on Safety of Secondhand Smoke Exposure from Tobacco or Cannabis

How safe is it to expose adults to 2nd hand smoke from tobacco or cannabis?

	**Tobacco (n = 5032)**				**Cannabis (n = 5002)**				
	**2020**	**2021**	**2021-2020**	**p-value**	**2017**	**2020**	**2021**	**2017-2021**	**p-value**
	n (%)	n (%)	Δn (Δ%)		n (%)	n (%)	n (%)	Δn (Δ%)	
**Completely unsafe**	4015 (80)	3960 (79)	-55 (-1)	.10	2583 (52)	2465 (49)	2528 (51)	-55 (-1)	<.001
**Somewhat unsafe**	917 (18)	953 (19)	36 (1)		1719 (34)	1878 (38)	1845 (37)	126 (3)	
**Somewhat safe**	71 (1)	79 (2)	8 (0)		482 (10)	482 (10)	473 (9)	9 (0)	
**Completely safe**	29 (1)	40 (1)	11 (0)		218 (4)	177 (4)	156 (3)	62 (-1)	

How safe is it to expose children to 2nd hand smoke from tobacco or cannabis?

	**Tobacco (n = 5032)**				**Cannabis (n = 5003)**				
	**2020**	**2021**	**2021-2020**	**p-value**	**2017**	**2020**	**2021**	**2017-2021**	**p-value**
	n (%)	n (%)	Δn (Δ%)		n (%)	n (%)	n (%)	Δn (Δ%)	
**Completely unsafe**	4416 (88)	4365 (87)	-51 (-1)	.04	3732 (75)	3781 (76)	3756 (75)	24 (0)	.13
**Somewhat unsafe**	541 (11)	577 (11)	36 (1)		991 (20)	988 (20)	1009 (20)	18 (0)	
**Somewhat safe**	53 (1)	55 (1)	2 (0)		196 (4)	165 (3)	171 (3)	-25 (-1)	
**Completely safe**	22 (0)	35 (1)	13 (0)		84 (2)	69 (1)	67 (1)	-17 (0)	

How safe is it to expose pregnant women to 2nd hand smoke from tobacco or cannabis?

	**Tobacco (n = 5031)**				**Cannabis (n = 5026)**			
	**2020**	**2021**	**2021-2020**	**p-value**	**2020**	**2021**	**2020-2021**	**p-value**
	n (%)	n (%)	Δn (Δ%)		n (%)	n (%)	Δn (Δ%)	
**Completely unsafe**	4435 (88)	4374 (87)	-61 (-1)	.10	3726 (74)	3678 (73)	-48 (-1)	.23
**Somewhat unsafe**	527 (10)	588 (12)	61 (1)		1024 (20)	1084 (22)	60 (1)	
**Somewhat safe**	49 (1)	51 (1)	2 (0)		186 (4)	184 (4)	-2 (0)	
**Completely safe**	20 (0)	18 (0)	-2 (0)		90 (2)	80 (2)	-10 (0)	

© 2023 Chambers J et al. *JAMA Network Open.*

eTable 5: Weighted Analyses for Analogous Question on Safety of Secondhand Smoke Exposure from Tobacco or Cannabis

How safe is it to expose adults to 2nd hand smoke from tobacco or cannabis?

	TOBACCO				CANNABIS				
	**2020**	**2021**	**2021-2020**	**p-value**	**2017**	**2020**	**2021**	**2017-2021**	**p-value**
	% (95% CI)	% (95% CI)	%		% (95% CI)	% (95% CI)	% (95% CI)	%	
**Completely unsafe**	79.9 (78.3–81.4)	78.6 (76.9–80.1)	-1.3	.21	50.9 (49.0–52.8)	47.9 (46.1–49.9)	49.2 (47.3–51.2)	-1.7	.02
**Somewhat unsafe**	18.0 (16.6–19.5)	18.7 (17.3–20.2)	0.7		33.1 (31.3–34.9)	36.1 (34.3–38.0)	35.3 (33.5–37.1)	2.2	
**Somewhat safe**	1.6 (1.2–2.3)	1.9 (1.4-2.5)	0.3		10.5 (9.4–11.8)	11.4 (10.2–12.8)	11.3 (10.1–12.6)	0.8	
**Completely safe**	0.5 (0.3–0.9)	0.9 (0.5–1.4)	0.4		5.5 (4.6–6.5)	4.4 (3.6–5.4)	4.2 (3.4–5.2)	-1.3	

How safe is it to expose children to 2nd hand smoke from tobacco or cannabis?

	**2020**	**2021**	**2021-2020**	**p-value**	**2017**	**2020**	**2021**	**2017-2021**	**p-value**
	% (95% CI)	% (95% CI)	%		% (95% CI)	% (95% CI)	% (95% CI)	%	
**Completely unsafe**	87.5 (86.2–88.7)	86.0 (84.6–87.3)	-1.5	.02	73.2 (71.4–74.5)	73.7 (72.0–75.4)	72.1 (70.3–73.9)	-1.1	.13
**Somewhat unsafe**	10.9 (9.7–12.1)	11.7 (10.5–13.0)	0.8		20.1 (18.6–21.7)	20.6 (19.1–22.2)	21.5 (20.0–23.2)	1.4	
**Somewhat safe**	1.2 (0.8–1.7)	1.5 (1.1–2.1)	0.3		4.4 (3.6–5.2)	4.0 (3.3–4.9)	4.6 (3.8–5.6)	0.2	
**Completely safe**	0.4 (0.2-0.8)	0.8 (0.5–1.3)	0.4		2.3 (1.7–3.1)	1.7 (1.2–2.3)	1.7 (1.3–2.4)	-0.6	

How safe is it to expose pregnant women to 2nd hand smoke from tobacco or cannabis?

	**2020**	**2021**	**2021-2020**	**p-value**	**2020**	**2021**	**2020-2021**	**p-value**
	% (95% CI)	% (95% CI)	%		% (95% CI)	% (95% CI)	%	
**Completely unsafe**	87.6 (86.3–88.9)	86.6 (85.2–87.9)	-1.0	.49	72.9 (71.1–74.5)	71.2 (69.4–72.9)	-1.7	.16
**Somewhat unsafe**	10.8 (9.6–12.0)	11.7 (10.5–13.0)	0.9		20.7 (19.2–22.2)	22.0 (20.5–23.7)	1.3	
**Somewhat safe**	1.2 (0.8–1.7)	1.2 (0.8–1.8)	0.0		4.1 (3.4–5.0)	4.8 (3.9–5.7)	0.7	
**Completely safe**	0.4	0.4	0.0		2.3	2.0	-0.3	

© 2023 Chambers J et al. *JAMA Network Open.*

	(0.2–0.8)	(0.2–1.0)	(1.7–3.1)	(1.5–2.7)

© 2023 Chambers J et al. *JAMA Network Open.*

<u>eTable 6:</u> Weighted Multivariable Analysis of Baseline Characteristics Predicting Change in Perception of Primary and Secondhand Smoke Exposure to Marijuana as Safer

How safe is smoking one marijuana joint a day versus one cigarette a day? N=3985*		
	**Adjusted OR (95% CI)**	**p-value**
**Age**		
18-29	1.4 (1.0 to 2.1)	.03*
30-44	1.0 (0.8 to 1.4)	.74
45-59	1.1 (0.9 to 1.4)	.39
60+	Ref	
**Marital status**		
Married	Ref	
Other	1.3 (1.1 to1.6)	.004*
**Working status**		
Working	Ref	
Retired	0.8 (0.6 to 1.0)	.06
Other	1.1 (0.9 to 1.5)	.42
**State baseline legal status**		
Non-legal	Ref	
Medical legal	1.2 (1.0 to 1.4)	.10
Recreational legal	1.0 (0.8 to 1.3)	.97

How does secondhand smoke from marijuana compare to secondhand smoke from tobacco? n=4110*		
	**Adjusted OR (95% CI)**	**p-value**
**Age**		
18-29	0.9 (0.8 to 1.4)	.68
30-44	0.9 (0.7 to 1.1)	.64
45-59	1.1 (0.8 to 1.2)	.70
60+	Ref	
**Working status**		
Working	Ref	
Retired	0.8 (0.6 to 1.0)	.09
Other	1.0 (0.8 to 1.3)	.93
**State baseline legal status**		
Non-legal	Ref	
Medical legal	1.2 (1.0 to 1.4)	.08
Recreational legal	1.0 (0.7 to 1.3)	.85

*Analyses exclude participants that already had the safest view of cannabis at baseline

© 2023 Chambers J et al. *JAMA Network Open.*

eTable 7: Multivariable Linear Analysis of Baseline Characteristics Predicting Change in Perception of Primary and Secondhand Smoke Exposure*

**How safe is smoking one marijuana joint a day versus one cigarette a day? n=3985***		
	**Adjusted Beta Coefficient (95% CI)**	**p-value**
**Age**		
18-29	0.22 (0.08 to 0.37)	0.002
30-44	0.02 (-0.09 to 0.14)	0.70
45-59	0.06 (-0.04 to 0.16)	0.21
60+	Ref	
**Gender**		
Male	Ref	
Female	0.08 (0.01 to 0.15)	0.03
**Income, n (%)**		
<$30,000	Ref	
$30,000-74,999	0.06 (-0.05 to 0.17)	0.29
$75,000-124,999	0.05 (-0.07 to 0.17)	0.40
>$125,000	0.06 (-0.07 to 0.18)	0.36
**Martial status**		
Married	Ref	
Other	0.12 (0.04 to 0.20)	0.003
**Working status**		
Working	Ref	
Retired	-0.15 (-0.25 to -0.05)	0.003
Other	0.02 (-0.09 to 0.14)	0.68
**State baseline legal status**		
Non-legal	Ref	
Medical legal	0.03 (-0.05 to 0.11)	0.50
Recreational legal	-0.06 (-0.15 to 0.03)	0.22
**How does secondhand smoke from marijuana compare to secondhand smoke from tobacco? n=4110***		
	**Adjusted Beta Coefficient (95% CI)**	**p-value**
**Age**		
18-29	0.08 (-0.0.6 to 0.21)	0.26
30-44	0.01 (-0.10 to 0.13)	0.84
45-59	0.07 (-0.3 to 0.17)	0.19
60+	Ref	
**Race/Ethnicity, n (%)**		
Black, non-Hispanic	-0.01 (-0.16 to 0.14)	0.90
Hispanic	-0.03 (-0.16 to 0.10)	0.62
Multiracial, non-Hispanic	0.10 (-0.12 to 0.33)	0.38
White, non-Hispanic	Ref	
Other, non-Hispanic	-0.02 (-0.19 to 0.15)	0.82
**Working status**		
Working	Ref	
Retired	-0.11 (-0.21 to -0.01)	0.03
Other	0.02 (-0.93 to 0.13)	0.73

*Positive numbers indicate a move towards a safer view of cannabis, negative numbers indicate a move towards a safer view of tobacco from 2017 to 2021.

© 2023 Chambers J et al. *JAMA Network Open.*

- All factors in Table 1 associated with change in perception in univariable models at p<.10 were included in multivariable models.
- Analyses exclude participants that already had the safest view of cannabis at baseline

© 2023 Chambers J et al. *JAMA Network Open.*
