# Supplementary material for: Perceptions of Safety of Daily Cannabis vs Tobacco Smoking and Secondhand Smoke Exposure, 2017-2021
Source: JAMA Netw Open. 2023 Aug 11;6(8):e2328691. doi: 10.1001/jamanetworkopen.2023.28691 (PMC10422186; doi:10.1001/jamanetworkopen.2023.28691)
Supplement: Supplement 2. — Data Sharing Statement [file jamanetwopen-e2328691-s002.pdf]

Data Sharing Statement

Chambers. Perceptions of Safety of Daily Cannabis vs Tobacco Smoking and Secondhand Smoke Exposure, 2017-2021. *JAMA Netw Open*. Published online August 11, 2023. doi:10.1001/jamanetworkopen.2023.28691

Data
Data available: No

Additional Information
Explanation for why data not available: Data dictionary and data to reproduce study findings can be obtained by contacting the corresponding author.
